# Novel variants in a patient with late-onset hyperprolinemia type II: diagnostic key for status epilepticus and lactic acidosis

**DOI:** 10.1186/s12883-019-1583-0

**Published:** 2019-12-29

**Authors:** Jeremias Motte, Anna Lena Fisse, Thomas Grüter, Ruth Schneider, Thomas Breuer, Thomas Lücke, Stefan Krueger, Huu Phuc Nguyen, Ralf Gold, Ilya Ayzenberg, Gisa Ellrichmann

**Affiliations:** 1 Department of Neurology, St. Josef-Hospital, Ruhr-University Bochum, Gudrunstrasse 56, 44791 Bochum, Germany; 2 Department of Internal Medicine, St. Josef-Hospital, Ruhr-University Bochum, Bochum, Germany; 3 University Children’s Hospital, St. Josef-Hospital, Ruhr-University Bochum, Bochum, Germany; 4 Center for Rare Diseases Ruhr (CeSER), Ruhr-University Bochum, Bochum, Germany; 5 Gemeinschaftspraxis für Humangenetik, Dresden, Germany; 6 Department of Human Genetics, Ruhr-University Bochum, Bochum, Germany; 7 Department of Neurology, Sechenov First Moscow State Medical University, Moscow, Russia

**Keywords:** Hyperprolinemia type II, *ALDH4A1* gene, Epilepsy, Vitamin B6 metabolism, Proline

## Abstract

**Background:**

Hyperprolinemia type 2 (HPII) is a rare autosomal recessive disorder of the proline metabolism, that affects the *ALDH4A1* gene. So far only four different pathogenic mutations are known. The manifestation is mostly in neonatal age, in early infancy or early childhood.

**Case presentation:**

The 64-years female patient had a long history of abdominal pain, and episode of an acute neuritis. Ten years later she was admitted into the neurological intensive-care-unit with acute abdominal pain, multiple generalized epileptic seizures, a vertical gaze palsy accompanied by extensive lactic acidosis in serum 26.0 mmol/l (reference: 0.55–2.2 mmol/l) and CSF 12.01 mmol/l (reference: 1.12–2.47 mmol/l). Due to repeated epileptic seizures and secondary complications a long-term sedation with a ventilation therapy over 20 days was administered. A diagnostic work-up revealed up to 400-times increased prolin-level in urine CSF and blood. Furthermore, a low vitamin-B_6_ serum value was found, consistent with a HPII causing secondary pyridoxine deficiency and seizures. The *ALDH4A1* gene sequencing confirmed two previously unknown compound heterozygous variants (*ALDH4A1* gene (NM_003748.3) Intron 1: c.62 + 1G > A - heterozygous and *ALDH4A1* gene (NM_003748.3) Exon 5 c.349G > C, p.(Asp117His) - heterozygous). Under high-dose vitamin-B_6_ therapy no further seizures occurred.

**Conclusion:**

We describe two novel *ALDH4A1*-variants in an adult patient with hyperprolinemia type II causing secondary pyridoxine deficiency and seizures. Severe and potentially life-threatening course of this treatable disease emphasizes the importance of diagnostic vigilance and thorough laboratory work-up including gene analysis even in cases with atypical late manifestation.

## Background

Hyperprolinemia type 2 (HPII) is an autosomal recessive disorder of the proline metabolism that is caused by a deficiency in pyrolin-5-carboxylate (P5C) dehydrogenase, which leads to an accumulation of P5C. In human, the protein is encoded by the *ALDH4A1* gene and only four different pathogenic mutations are known so far (HGMD® Professional 2019.1) [1]. Pyridoxal phosphate (PLP) (active vitamin-B_6_ coenzyme) is de-activated by P5C acid [2], and PLP-depended enzymatic reactions in amino acid and neurotransmitter metabolism are disturbed. Consequently, PLP utilization is increased [2]. Neither prevalence nor incidence of HPII are exactly known. A 18-years long screening of 20,991 urinary organic acid profiles from an academic referral center in the Netherlands estimated a cumulative incidence of HPII of approximately 1 in 700,000 newborns [3]. Manifestation of previously reported patients was mostly in neonatal age, in early infancy or early childhood [4]. Patients suffered from generalized epileptic seizures and intellectual disability [4, 5].

## Case presentation

In 2006, a 52-year old female patient was admitted to a neurological department due to sudden difficulties with swallowing and speech, ophthalmoparesis with a vertical and horizontal eye movement disorder, dysesthesia of the hands with a quality of “pins and needles” and a glove-like distribution, as well as generalized areflexia. Muscle strength was normal. A Miller Fisher syndrome was diagnosed. Under treatment with 150 g intravenous immunoglobulins the symptoms completely remitted within a few days. In the following years, the patient consulted the general practitioner and several gastroenterologists because of unspecific abdominal pain, from which she has been suffering since childhood. Diagnostics including computer tomography (CT), magnetic resonance imaging (MRI) and gastroscopy were normal.

In October 2017 the meanwhile 63-year old patient was hospitalized with generalized epileptic seizures with prolonged postictal confusion. Cerebral MRI showed no pathological findings, and therapy with levetiracetam was started.

In December 2017 the patient was admitted to our clinic for internal medicine because of persisting diarrhea, abdominal pain, renal failure and a reduced general state of health. Again, the patient had generalized epileptic seizures with postictal confusion and significantly reduced vigilance, accompanied from a lactic acidosis (serum-lactate 26.0 mmol/l (reference 0.55–2.2 mmol/l), pH 6.863), leading to admission to the neurological intensive care unit. Moreover, a slight, presumably residual ophthalmoparesis as vertical gaze palsy with conjugate, bilateral limitation of the eye movements in upgaze was evident. The anticonvulsive therapy with levetiracetam (3 g/day) was extended by lacosamide (400 mg/day). A mechanical ventilation was necessary due to the sudden and massive metabolic acidosis as well as a respiratory failure during an epileptic seizure (serum-lactat 14.7; 10.6; 16.0 mmol/l). In CSF, lactate (12.01 mmol/l (reference 1.12–2.47 mmol/l) and protein level (67 mg/dl (reference 15–45 mg/dl) were significantly increased. There were no signs for an infectious origin in CSF (polymerase chain reaction for neurotrophic germs, including Tropheryma whippelii). CT and MRI of the brain as well as abdominal- and thorax-CT were normal. The electroneurography revealed a slight mixed axonal-demyelinating polyneuropathy, the electromyography was normal. Besides a sinus-tachycardia with 140 bpm and a mild pericardial effusion, no signs of a Wolff–Parkinson–White syndrome, that would be common in mitochondriopathies, were present.

Attempted extubation failed as another severe epileptic seizure occurred with life-threatening lactic acidosis and hyperkalemia (lactate 26.0 mmol/l; pH 6.925; potassium 7.8 mmol/l (referece 3.6–5.2 mmol/l). EEG showed an alpha rhythm, with intermittent slow waves and tendency to generalize. In cerebral follow-up MRI, multiple fat embolies were detected. CT-angiography of the lung revealed a pulmonary embolism. As a reason for the fat embolies, multiple vertebral fractures were verified in CT, presumably as a result of severe epileptic seizures. A surgical fixation of vertebral fractures was performed. Intensive care therapy including ventilation was necessary for more than eight weeks. Weaning was successful after dilatative tracheostomy and nutrition via percutaneous endoscopic gastrostomy (PEG) tube. Vitamin-B_6_ was supplemented. Subsequently, a rehabilitative therapy was performed. After five months, the patient returned to her normal life. Tracheostomy and PEG were removed.

Taking into account all the symptoms (abdominal pain, relapsing remitting course, neurological deficits, epileptic seizures, peripheral neuropathy, lactic acidosis, cardiac disturbance), two main differential diagnosis were discussed: porphyria and a mitochondrial disease (MERRF syndrome). None of both diagnoses could be verified: Laboratory test revealed no hints of porphyria (Porphyria Specialist Center of the European Porphyria Network University Hospital Düsseldorf, Germany). A muscle biopsy gave no hint of a mitochondrial disease and revealed only a slight unspecific atrophy that was very likely due to immobility (Institute of Neuropathology of the University Hospital Essen, Germany). In terms of differential diagnosis, other metabolic diseases came into consideration. Comprehensive diagnostic for diseases of copper metabolism, lead poisoning or adrenoleukodystrophy remained inconspicuous. Finally, the analysis of the amino acids in the urine, CSF and serum showed a strong abnormality with ubiquitously increased amino acids, especially proline (proline in serum 3085 μmol/l (reference 90-342 μmol/l), in urine 46,531 μmol/g Crea (reference < 100 μmol/g Crea), in CSF 104 mg/dl (reference < 6 mg/dl), Hydroxyproline in urine 1395 μmol/g Crea (reference <100) Table 1). Furthermore, vitamin-B_6_ was decreased with 3.3 μg/l (reference > 4.9 μg/l). We supplemented vitamin B_6_ with 200 mg/d.

**Table 1:** Amino acids in blood, urine and CSF

Amino acid	blood μmol/l	reference μmol/l	urine μmol/g Crea	reference μmol/g Crea	CSF mg/dl	Reference mg/dl
Alanine	685	205–508	929.0	< 700	32	19–60
Arginine	113	40–140	< 9.0	< 150	18	11–32
Asparagine	90	39–79	59.0	< 500	10	5–20
Aspartic acid	51	< 35	352.0	< 100	< 3	< 3
Cysteine			31	< 200	4	< 3
Glutamine	284	470–758	383.0	< 800	466	380–1348
Glutamine acid	420	28–92	25.	< 200	< 3	< 4
Glycine	710	120–387	19,664.0	< 2500	12	< 35
Histidine	118	30–120	781.0	< 1600	16	9–28
**Hydroxyproline**	**–**	**–**	**1395**	**< 100**	**–**	**–**
Isoleucine	68	35–100	19.0	< 100	< 3	< 17
Lysine	217	82–260	401.0	< 250	21	13–42
Methionine	23	6–40	56.0	< 100	< 3	< 10
Phenylalanine	120	37–115	160	< 150	7	7–21
**Proline**	**3085**	**90–342**	**46,531**	**< 100**	**104**	**< 6**
Serine	226	67–193	12.0	< 800	31	19–40
Threonine	152	75–194	25	< 500	36	23–57
Tryptophan	50.0	34–90	15.0	< 150	< 3	< 6
Tyrosine	86.0	21–107	386.0	< 200	13	5–17
Valine	198	116–317	77.0	< 120	9	11–40

Values were obtained in a fasted state in the morning.

Significant data are set in Bold

**Fig. 1:**
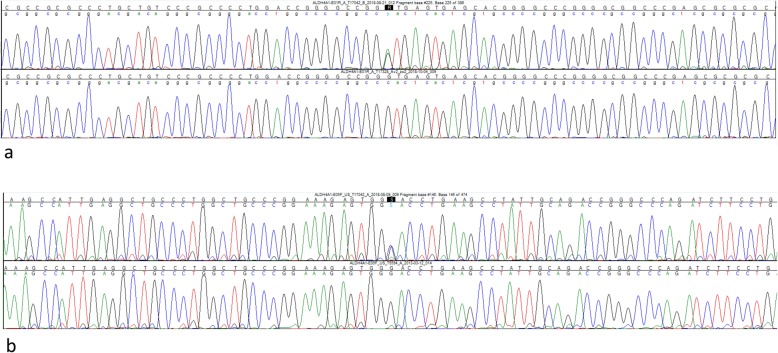
First variant (**a**): *ALDH4A1* gene (NM_003748.3) Intron 1: c.62 + 1G > A heterozygous. This variant affects a canonical nucleotide of the splice donor site of intron 1 and is therefore thought to lead to aberrant splicing. Second variant (**b**): *ALDH4A1* gene (NM_003748.3) Exon 5 c.349G > C, p.(Asp117His) heterozygous. This missense variant affects a highly conserved residue in the dehydrogenase domain of the protein. Furthermore, three out of four in silico predictions applied (SIFT, MutationTaster, Polyphen-2) support the role of this sequence alteration as a pathogenic variant

Therefore, a hyperprolinemia type I or type II was most likely. The targeted genetic analyses by Sanger sequencing revealed no pathogenic variant within the *PRODH*-gene (hyperprolinemia type I) but identified two novel variants within the *ALDH4A1*-gene (Fig. 1). In combination, both heterozygous variants within the *ALDH4A1* gene could lead to a compound heterozygosity (variants *in trans*), that would cause the described disease phenotype. Since the patient’s son had only one of the two heterozygous variants of his mother, the compound heterozygous state of both variants in our patient was proven.

## Discussion and conclusions

We describe a case of HPII with two novel *ALDH4A1*-variants in a compound heterozygous state. The first variant within the *ALDH4A1* gene affects a canonical nucleotide of the splice donor site of intron 1 and is therefore thought to lead to aberrant splicing. The first variant is according to the Plon 5-step classification a class 4 variant (probably pathogenic).

The second variant within the *ALDH4A1* gene is a missense variation and affects a highly conserved residue in the dehydrogenase domain of the protein. Furthermore, three out of four in silico predictions applied (SIFT, MutationTaster, Polyphen-2) support the role of this sequence alteration as a pathogenic variation. This variant is according to the Plon 5-step classification a class 3 variant (possibly pathogenic). In summary, it is highly probable that the two identified *trans-ALDH4A1* variants are causally responsible for the disease.

The unique of this case is the rareness of HPII and the novel variants within the *ALDH4A1* gene, with an adult and fulminant disease onset. In the late 1980s, Flynn et al. [6]. showed a cohort of 312 Irish travelers in which 14 HPII patients were reported. There were 7 healthy adult patients with HPII, the oldest with an age of 36 years.

Despite evidence of the genetic alterations, the exact pathomechanism remains unclear. Malnutrition and liver diseases can be a cause of hyperprolinaemia, however this patient did not show any signs of it.

The sudden critical deterioration of the patient’s condition seems to be the result of a massive acidosis. A lactic acidosis can lead to secondary hyperprolinemia [7]. and thus be a potential trigger for deterioration. Vice versa, the mutated P5C dehydrogenase is a mitochondrial enzyme and a lactic acidosis could be result of the gene defect [8]. Our suggestion however is, that the heterozygote patient’s gene variations lead to a latent hyperprolinaemia. Because of abdominal pain and a general deterioration of condition the patient developed an increase of lactate and an increase of proline. Proline accumulation results in oxidative stress and reduced Na^+^+K^+^-ATPase activity which led to a circulus virtuoso with further lead to mitochondrial stress and resulted in increasing lactate levels.

P5C is a unique endogenous vitamin-B_6_ antagonist. The inactivation of vitamin-B_6_ by P5C may contribute to seizures in HPII [7]. In rat brain acute and chronic hyperprolinemia reduced glutamate uptake, Na^+^- K^+^-ATPase activity and ATP levels [9], which could be a reason for seizures. Long-term vitamin B6 supplementation may prevent these seizures [2]. However, in a Dutch cohort the clinical course of HPII was non-progressive and independent from the B_6_ concentration and B_6_ therapy [3]. Whether the vitamin B_6_ deficiency, detected in our patient was ultimately responsible for the seizures remains unclear. However, the previous case reports suggest such a pathomechanism, and the supplementation with vitamin B_6_ led to a seizure-free period.

According to previous publications, individuals with hyperprolinemia should be monitored intensively [3]. If acute manifestations of hyperprolinemia such as epileptic seizures are controlled, the prognosis for these disorders is quite good [7].

In conclusion, to our knowledge this is the first description of an adult patient showing two novel heterozygous variants within the *ALDH41* gene in a compound heterozygous state. The late onset relapsing-remitting, potentially life-threatening course of this treatable disease emphasizes the importance of diagnostic caution and thorough laboratory work-up in cases with atypical clinical presentations. Metabolic diseases are often a chameleon and must always be considered in the differential diseases of the nervous system. Especially in therapy refractory seizures, not only in children, a hyperprolinemia causing secondary pyridoxine deficiency and seizures should be considered.

## Data Availability

The datasets used and/or analysed during the current study are available from the corresponding author on reasonable request.

## Abbreviations

*ALDH4A1*-gene: Aldehyde dehydrogenase 4 family, member A1 - gene
Asp: Aspartic acid
CSF: Cerebrospinal fluid
CT: Computer tomography
EEG: Electroencephalography
G > A: Guanine to Adenine
G > C: Guanine to Cytosine
HGMD: Human Gene Mutation Database
His: Histidine
HPII: Hyperprolinemia type 2
MERRF-syndrome: Myoclonic epilepsy with ragged red fiber syndrome
MRI: Magnetic resonance imaging
P5C: Pyrolin-5-carboxylate
PEG: Percutaneous endoscopic gastrostomy
pH: Potentia Hydrogenii
PLP: Pyridoxal phosphate
*PRODH*-gene: Proline Dehydrogenase 1 – gene
SIFT: Sorting Intolerant From Tolerant (Database)
